# Draft Genome Sequences of Two *Streptococcus pneumoniae* Serotype 19A Sequence Type 226 Clinical Isolates from Hungary, Hu17 with High-Level Beta-Lactam Resistance and Hu15 of a Penicillin-Sensitive Phenotype

**DOI:** 10.1128/genomeA.00401-17

**Published:** 2017-05-18

**Authors:** Martin Rieger, Dalia Denapaite, Reinhold Brückner, Patrick Maurer, Regine Hakenbeck

**Affiliations:** Department of Microbiology, University of Kaiserslautern, Kaiserslautern, Germany

## Abstract

The draft genome sequences of two multiple-antibiotic-resistant *Streptococcus pneumoniae* isolates from Hungary, Hu15 and Hu17, are reported here. Strain Hu15 is penicillin susceptible, whereas Hu17 is a high-level-penicillin-resistant strain. Both isolates belong to the serotype 19A sequence type 226, a single-locus variant (in the *ddl* locus) of the Hungary^19A^-6 clone.

## GENOME ANNOUNCEMENT

High-level-penicillin- and multiple-antibiotic-resistant *Streptococcus pneumoniae* (PRSP) strains of serotype 19A were prevalent in Hungary during the 1990s (1, 2). The strain HUN663 represents the clone Hungary^19A^-6, as defined by multilocus sequence typing (MLST), belonging to sequence type 268 (ST268) (3). Meanwhile, another 11 strains representing 4 single-locus variants (SLVs) of Hungary^19A^-6 with respect to the *ddl* locus are reported in the MLST database (http://pubmlst.org/spneumoniae/), including isolates from the Czech Republic and Slovakia (4) and Norway belonging to ST226, ST340, ST382, and ST7133. The *ddl* locus maps closely to *pbp2b*, which is acquired by horizontal gene transfer during the evolution of penicillin resistance (5) and is therefore not used in phylogenetic analyses based on MLST. Therefore, all these strains can be considered to belong to the clone Hungary^19A^-6. In agreement, strains of ST258 and ST226 express a penicillin-binding protein 3 (PBP3) of different electrophoretic mobility compared to that of most other *S. pneumoniae* (1, 6, 7) strains. 

Isolates of ST226 are varied in their MIC values, PBP profile, and PBP2x sequences, and multilocus electrophoretic typing revealed several electrophoretic types (6, 8). Accordingly, their genomes appear surprisingly variable compared to other clones (9); in fact, Hungary^19A^-6 had acquired the largest proportion of genes (8.2%) from *Streptococcus mitis* in a comparative genomic analysis of 35 *Streptococcus* species genomes (10). These strains are part of the Kaiserslautern strain collection obtained from Anna Marton as cited in (6) now held at the German National Reference Center for Streptococci in Aachen, Germany. Interestingly, one member (strain Hu15) was penicillin susceptible (MICs, 0.1 µg/ml for oxacillin and 0.024 µg/ml for cefotaxime), whereas Hu17 is among the strains expressing the highest beta-lactam resistance levels (MICs, 30 and 1.6 µg/ml, respectively). Moreover, the strains were resistant to tetracycline, streptomycin, erythromycin, and trimetroprim.

Information retrieved from these two genomes will help decipher the evolutionary pathway of penicillin resistance. The genome sequences revealed a plasmid related to pSpnP1 (11).

The genomes were sequenced using an Illumina HiSeq platform (Hu15/Hu17, 2,203,997/2,192,370 bp of paired-end reads). Genomes were assembled using gsAssembler (version 2.6). RATT was used for genome annotation (12) using *S. pneumoniae* Hu19A-6 as a reference genome, and was adjusted manually if necessary, according to NCBI submission guidelines. The five capped small RNA (csRNA) genes encoding small regulatory RNAs controlled by CiaRH (13) were identified by their high identity to the *S. pneumoniae* R6 counterparts and added to the annotation. The genomes of Hu15/Hu17 were assembled into 176/200 contigs, with a total length of 2,136,165/2,141,026 nucleotides (nt) (sequenced to ~2.2 million reads with ~157/159× coverage). A plasmid was assigned to 1/4 contig(s), with a total length of 5,327/5,112 nt. The predicted genes from the genomes include 2,191/2,190 coding sequences (CDSs), 64/93 incomplete genes at contig ends, 2/3 rRNAs, 22/23 tRNAs, 5/4 csRNAs, and 3/3 other small RNAs (RNase P; *srpB*; transfer-messenger RNA [tmRNA]).

### Accession number(s).

The draft genome sequences and plasmid sequences of Hu15 and Hu17 have been deposited in the NCBI database under the GenBank accession numbers CP020551 and CP020552 and CP020549 and CP020550, respectively.
